# The rates and medical necessity of cesarean delivery in China, 2012–2019: an inspiration from Jiangsu

**DOI:** 10.1186/s12916-020-01890-6

**Published:** 2021-01-25

**Authors:** Ci Song, Yan Xu, Yuqing Ding, Yanfang Zhang, Na Liu, Lin Li, Zhun Li, Jiangbo Du, Hua You, Hongxia Ma, Guangfu Jin, Xudong Wang, Hongbing Shen, Yuan Lin, Xiaoqing Jiang, Zhibin Hu

**Affiliations:** 1 State Key Laboratory of Reproductive Medicine, Nanjing Medical University, Nanjing, 211166 China; 2 Department of Epidemiology, School of Public Health, Nanjing Medical University, Nanjing, 211166 China; 3 Department of Maternal and Child Health, Jiangsu Commission of Health, Nanjing, 210008 China; 4 Department of Group Health, Women and Children Branch Hospital of Jiangsu Province Hospital/Jiangsu Women and Children Health Hospital, Nanjing Medical University, Nanjing, 210036 China; 5 Department of Social Medicine & Health Education, Nanjing Medical University, Nanjing, 211166 China; 6 Department of Maternal, Child and Adolescent Health, School of Public Health, Nanjing Medical University, Nanjing, 211166 China

**Keywords:** Cesarean delivery, Rate, Medical necessity, China

## Abstract

**Background:**

The World Health Organization (WHO) in 2015 stated that every effort should be made to provide cesarean delivery (CD) for women in need. In China, the two-child policy largely prompts the number of advanced age childbirth, which raises the possibility of an increasing number of women who need a c-section. The aim of this study was to assess the trends in the overall and medical indication-classified CD rates in the era of the two-child policy in Jiangsu, China.

**Methods:**

A retrospective cross-sectional study of 291,448 women who delivered in 11 hospitals in Jiangsu province between 2012 and 2019 was conducted. Medical cesarean indication for each woman was ascertained by manually reviewing the medical records. The 291,448 women were divided into two subgroups according to the presence of the indications: the indicated group (7.80%) and the non-indicated group (92.20%). We then fitted joinpoint regression and log-binomial regression models to estimate trends in the CD rates across the study period.

**Results:**

The overall CD rate was observed with a declining trend from 52.51% in 2012–2015 to 49.76% in 2016–2019 (adjusted RR, 0.92; 95% CI, 0.91–0.93; *P* < 0.001), along with an annual percentage change (APC) to be − 1.0 (95% CI, − 2.1 to 0.0) across the period. The participants were then divided into two subgroups according to the presence of medical CD indications: the indicated group (7.80%) and the non-indicated group (92.20%).We found the declining trend was most pronounced in the non-indicated group, with the CD rates decreased from 50.02% in 2012–2015 to 46.27% in 2016–2019 (adjusted RR, 0.90; 95% CI, 0.89–0.90; *P* < 0.001). By contrast, we observed a steady trend in the CD rate of the indicated group, which maintained from 87.47% in 2012–2015 to 86.57% in 2016–2019 (*P* = 0.448). In the indicated group, a higher risk of adverse pregnancy outcomes was revealed for those women who delivered vaginally as compared with those who received c-section. We further investigated that women with following specific indications had a higher proportion of vaginal delivery, i.e., pregnancy complications, fetal macrosomia, and pregnancy complicated with tumor (34.70%, 10.84%, and 16.34%, respectively). Women with the above 3 indications were observed with a higher risk of adverse pregnancy outcomes if delivered vaginally. The incidence rates of the medical indications among the general population increased considerably over the 8-year period (*P* < 0.001).

**Conclusions:**

Although the overall CD rate apparently decreased in the recent years, along with the decline of the unnecessary CD rate, a considerable proportion of indicated women were not provided with CD service in Jiangsu, China. Instead of targeting the overall CD rate, we need to take actions to reduce unnecessary CD rate and provide adequate c-section service for women with indications, particularly for those with underlying diseases and suspected fetal macrosomia.

**Supplementary Information:**

The online version contains supplementary material available at 10.1186/s12916-020-01890-6.

## Background

In 1985, the World Health Organization (WHO) has considered the ideal rate for cesarean delivery (CD) to be 10–15%. In 2015, George Molina et.al. proposed that the ideal CD rates of up to 19% for lower maternal or neonatal mortality [1]. Concerning about the rise in the numbers of CD worldwide since then [2], WHO conducted two worldwide studies to revisit the 1985 recommended rate. Based on the analysis, the new statement from WHO in 2015 concluded that every effort should be made to provide CD to women in need, rather than striving to achieve a specific rate [3–5]. Affording adequate service for medically indicated CD and avoiding medically unnecessary operations is the method to minimize the short and long term risk.

The CD rate in China was alarming since WHO reported that 46.2% of births were delivered by cesarean in 2007–2008, with an 11.7% rate of operations performed without medical indication, the highest in the world [6]. The national overall annual CD rate increased from 2008 to 2014 [7]. The potential risk of CD has prompted some developed areas to investigate strategies and implement interventions to reduce CD rates [8, 9]. As a result of the efforts, the overall CD rates declined in the last several years in some of the largest urban areas in China [7, 10]. The primary contributor to this decreasing trend was the reduction in the CD on maternal request rate (CDMR) [11], which is the main component of non-indicated CD. Additionally, China’s universal two-child policy was announced in October 2015, which targeted women of reproductive age who had a previous delivery [12]. Considering the possible adverse consequences of c-section on future pregnancy, primiparous women might be less likely to opt for this procedure when giving first birth after the implication of the policy. Meanwhile, the incidence of pregnancy complications grows rapidly with the rise of maternal age after the implementation of the policy in the recent years [12], which arouses the possibility of an increasing number of women meeting the medical indications for CD. Limited studies described the rate of medically indicated CD in the era of the two-child policy.

We aimed to determine the trends in the overall and indication-specific CD rates between 2012 and 2019 in Jiangsu, China, and to further assess the medical necessity of CD.

## Methods

### Data source

We conducted a cross-sectional study using individual level data collected by the National Maternal Near Miss Surveillance System (NMNMSS) of Jiangsu province covering in-hospital births from 11 hospitals between January 1, 2012, and December 31, 2019 [13, 14]. Briefly, the surveillance sites within the province were stratified by region and urban or rural characteristics, and the selected districts or counties were sampled randomly within strata to ensure proportional representation of urban and rural populations across the province. Within each of the sampled districts or counties, two health facilities with more than 1000 deliveries per year were randomly selected (or one facility if only one was available). As a result, 11 hospitals located in four urban districts and four rural counties in eastern, western, northern, and southern Jiangsu province were enrolled (Additional file 1: Figure S1). Doctors at each facility were trained to collect health-related data from admission to discharge and to complete a specially designed data collection form for each woman. Every hospital has at least one doctor assigned to the NMNMSS network. The individual level data includes date of delivery, maternal age, parity, delivery mode, level of education, hospital level, birth plurality, and discharge diagnosis.

### Data extraction and cleaning

Between January 1, 2012, and December 31, 2019, we extracted 324,198 unrepeated individual level records from the 11 hospitals in the Jiangsu NMNMSS database. Mothers left hospitals before delivery, with missing data for parity or birth outcomes, incorrect data for maternal age at delivery (age < 15.0 or ≥ 50.0), delivered at gestational age < 28 weeks, and had an abortion were excluded in our study. In addition, women who had antepartum fetal death were excluded, because the birth outcomes were acquainted before entering into the labor course (Additional file 2: Figure S2).

### Definition of variables

Delivery mode was categorized as vaginal delivery and CD. Maternal age was grouped in the following categories: 15–24 (reference category), 25–29, 30–34, and ≥ 35. This categorization is commonly used in the literature; maternal age ≥ 35 years is generally considered advanced maternal age. Delivering hospital was categorized as tertiary-level A grade (grade 3A), tertiary-level B grade (grade 3B), and secondary-level A grade (grade 2A) according to the Measures for the Administration of the Hospital Grade. Secondary hospitals are responsible for providing comprehensive health services and medical education and conducting research on a regional basis, which tend to be affiliated with medium sized urban or rural areas and contain 100~500 beds. Tertiary hospitals are comprehensive or general hospitals at the city, provincial, or national level with a bed capacity exceeding 500. They are responsible for providing specialist health services, which perform a bigger role with regard to medical education and scientific research. Of which, grade 3A hospitals are the top level, which provide high-quality medical service to citizens from different areas and conduct academic research and higher education. Discharge diagnosis and procedure codes were used to identify medical cesarean indication. Medical cesarean indication was defined on the basis of the Expert Consensus on Cesarean section Surgery developed by the Obstetrics Branch of Chinese Medical Association [15]. Medical cesarean indications were recognized if the following clinical criteria were present (Additional file 3: Table S1) in the discharge diagnosis: (1) fetal distress; (2) cephalopelvic disproportion (CPD); (3) scar uterus; (4) abnormal fetal position, e.g., transverse presentation, primiparous singleton term breech delivery and foot presentation; (5) placenta previa and vasa previa; (6) twin or multiple pregnancies, e.g., firstborn non-cephalic position, complex twin pregnancy, conjoined twins, and multiple pregnancies; (7) omphaloproptosis; (8) placental abruption; (9) severe pregnancy complications, e.g., acute cardiac morbidity (acute myocardial infarction, cardiac arrest, heart failure, peripartum cardiomyopathy, congenital heart disease), respiratory morbidity (respiratory failure, respiratory insufficiency, pulmonary hypertension, pulmonary embolism, pulmonary edema), gestational hypertension (severe preeclampsia, eclampsia, HELLP), renal failure, liver failure, acute fatty liver in gestation, severe intrahepatic cholestasis during pregnancy, severe anemia (hemoglobin < 60 g/L), thrombocytopenia, and cerebrovascular morbidity (cerebral infarction, intracranial hemorrhage, cerebral venous thromboembolism); (10) fetal macrosomia; (11) birth canal malformation, e.g., uterine malformation, uterine mediastinum, uterus duplex, bicornuate uterus, rudimentary uterus, transvaginal septum, double vagina, scar stricture of vagina, and pelvic stenosis; (12) varicose vulvar veins; (13) genital tract infects, gonorrhea, and condyloma acuminatum; (14) combined with tumor, e.g., malignant tumor of other system, cervical cancer, cervical myoma, and hysteromyoma.

Stillbirth was defined as the baby death at or after 28 weeks of gestation during delivery (antepartum fetal death was excluded in this study). Neonatal death was defined as infant death within 28 days after birth. Perinatal mortality was defined as stillbirth or neonatal death. Low birth weight describes babies who were born weighing less than 2500 g. In addition, as the 1 min and 5 min Apgar scores could indicate the severity of asphyxia to some extent, Apgar scores were also included as a birth outcome in our study.

### Analysis

#### Displaying temporal patterns of the overall and cesarean indication-specific CD rates

We enumerated the number by each medical cesarean indication criteria and calculated the overall CD rate. Women with two or more medical cesarean indications appear more than once in Additional file 3: Table S1. A connected-line plot of the overall and parity-specific CD rates by year was generated from January 2012 to December 2019. Joinpoint regression models were used to estimate trends in overall CD rates from 2012 to 2019 and to estimate annual percentage change (APC). The same models were used to test the differences across parity subgroups. Joinpoint regression analysis identified time points in which trends significantly change (i.e., joinpoints), using calendar year as the timescale. If the APC was non­significant (*P* ≥ 0.05), we regarded trends as stable; otherwise, the rate increased or decreased from 2012 to 2019. The period 2012–2019 was then divided into 2 stages at 2016 to visualize change patterns in long-term trends. Adjusted risk ratios (RRs) and 95% confidence intervals (CIs) analyzed by log-binomial regression models were used to identify the change pattern in overall and parity-specific CD rates after 2016. The descriptive analyses were then separately performed in subgroups of women with/without medical cesarean indications. In this study, required CD rate refers to the CD rate of women with medical cesarean indications; nonessential CD rate refers to the CD rate of women without medical cesarean indications.

#### Evaluating the medical necessity of CD among women with cesarean indications

Adjusted RRs and 95% CIs analyzed by log-binomial regression models were used to estimate the association between delivery mode (vaginal delivery/CD) with the risks of adverse pregnancy outcomes, including stillbirth, neonatal death, low Apgar score at 1 min and 5 min. Conventional covariates were parity, maternal age (15~24 years, 25~29 years, 30~34 years, ≥ 35 years), level of delivering hospital (grade 3A, grade 3B, grade 2A), and education level (primary school or illiteracy, middle school, high school, graduate or above).

#### Assessing the risk of adverse pregnancy outcomes conferred by unrecommended delivery mode by specific medical indication

To identify which medical cesarean indications were prone to end up with unrecommended delivery mode, we compared the CD rate among each cesarean indication-subgroup. The *χ*^2^ test was performed to examine if vaginal delivery rate was significant higher in any specific indication group. Then, we investigated the association between the risk of adverse pregnancy outcomes with unrecommended delivery mode in individual indication groups using adjusted RRs and 95% CIs analysis by log-binomial regression models. Furthermore, we displayed annual incidence rate of individual CD indications and generated a connected-line plot by year for the incidence rate from 2012 to 2019 for the indications showed high proportion of unrecommended delivery mode or conferred significant risk for adverse pregnancy outcomes. All analyses were conducted using STATA/SE 14.0.

#### Patient and public involvement

Neither patients nor the public were involved in the study design, data analysis, or interpretation of the study results. The results of this study will be disseminated via the media center of the authors’ institutions and probably also via mass media.

## Results

### The nonessential CD rate significantly decreased while the required CD rate unchanged

**Fig. 1:**
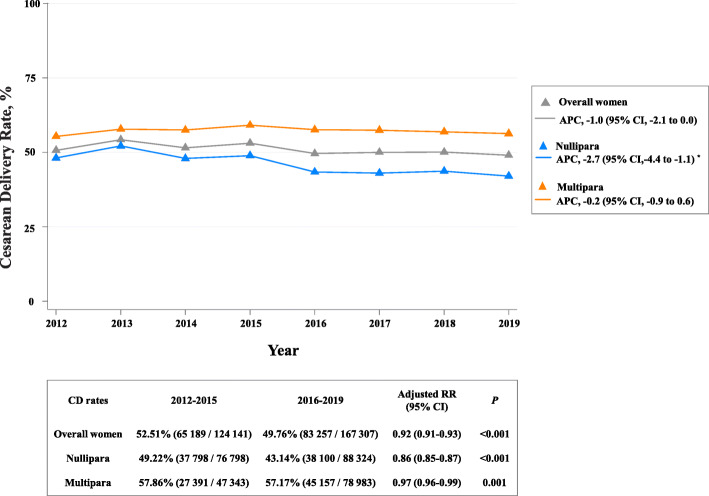
Secular trends of the overall and parity-specific CD rates from 2012 to 2019, by year. Gray triangles: annual CD rates for all births; blue triangles: annual CD rates for nulliparas; orange triangles: annual CD rates for multiparas. Annual percentage change (APC) of the CD rates estimated by joinpoint regression models was shown in the right gray frame. The CD rates before and after 2016 were listed in the below gray frame, along with the adjusted RRs and 95% CI represented the change pattern after 2016

A total of 315,576 records with obstetric outcomes were collected between January 1, 2012, and December 31, 2019. We then excluded records with missing or likely incorrect data for maternal age at delivery (age < 15.0 or ≥ 50.0, *n* = 8322, 2.57%), parity (*n* = 38, 0.01%), birth outcomes (*n* = 1331, 0.41%), births before 28 weeks' gestation (*n* = 13,162, 4.06%), had an abortion (*n* = 512, 0.16%), or antepartum fetal death (*n* = 763, 0.24%). The remaining 291,448 births were included in the study (Additional file 2: Figure S2). Among the 291,448 women, 23,405 (8.03%) were 35 years or older, 126,326 (43.34%) were multiparous, 60,594 (20.79%) delivered in grade 2A hospitals, and 3477 (1.20%) had an education level of primary school or illiteracy. The overall CD rate was 50.93% (148,446). Figure 1 depicted the declined secular trend for the overall CD rate from 2012 to 2019. Joinpoint regression estimated the overall APC to be − 1.0 (95% CI, − 2.1 to 0.0) between 2012 and 2019. After adjustments for parity, maternal age, level of delivering hospital, and education level, the overall CD rates decreased from 52.51% in 2012–2015 to 49.76% in 2016–2019 (adjusted RR, 0.92; 95% CI, 0.91–0.93; *P* < 0.001). The declining trend was most notable among nulliparous women, with the APC of − 2.7 (95% CI, − 4.4 to 1.1) between 2012 and 2019 (*P* for joinpoint regression < 0.001). The CD rates significantly decreased from 49.22% in 2012–2015 to 43.14% in 2016–2019 (adjusted RR, 0.86; 95% CI, 0.85–0.87; *P* < 0.001).

According to the assessment standards, we categorized women with one or more medical cesarean indications into the indicated group, which accounted for 7.80% (22,741/291,448) of the total women, and those who had no indications into non-indicated group (92.20%) (Additional file 4: Table S2). Compared with the non-indicated group, women in the indicated group were more likely to be of advanced age (the proportion of women aged ≥ 35 was 14.88% in the indicated group vs. 7.45% in the non-indicated group), more likely to be multiparas (51.50% vs. 42.65%), and delivered in the grade 3A hospitals (59.70% vs. 48.56%). The majority of the indicated group had a CD (86.90% vs. 47.89% in the non-indicated group). In other words, 13.10% of women who had medical cesarean indications delivered vaginally.

**Fig. 2:**
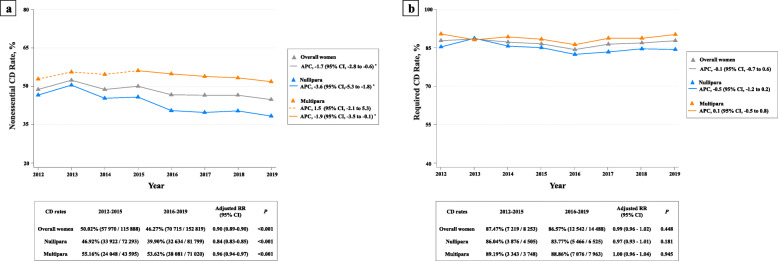
**a** Secular trends of the nonessential CD rates (the CD rates among women without medical indications) from 2012 to 2019, by year. **b** Secular trends of the required CD rates (the CD rates among women with medical indications) for women with indications from 2012 to 2019, by year

The CD rates for women who had medical cesarean indications (defined as required CD rate) and those who had no indications (defined as nonessential CD rate) were shown in Fig. 2. The declining tendency of the overall CD rates was mainly attributed by the decrease of the nonessential CD rates in women who had no medical indications. APC of the nonessential CD rates was estimated to be − 1.7 (95% CI, − 2.8 to − 0.6) between 2012 and 2019 (*P* < 0.001). The nonessential CD rate decreased from 50.02% in 2012–2015 to 46.27% in 2016–2019 (adjusted RR, 0.90; 95% CI, 0.89–0.90; *P* < 0.001; Fig. 2a). Interestingly, the required CD rates showed quite another trend throughout the study period. The required CD rate showed a steady trend from 2012 to 2019, which maintained from 87.47% (7219/8253) in 2012–2015 to 86.57% (12,542/14,488) in 2016–2019 (adjusted RR, 0.99; 95% CI, 0.96–1.02; *P* = 0.448; Fig. 2b). The APC for the required CD rates between 2012 and 2019 was − 0.1 (95% CI, − 0.7 to 0.6). This steady trend was consistently observed across subgroups stratified by parity (Fig. 2b) as well as level of delivering hospital (Additional file 5: Figure S3).

### Cesarean delivery was necessary among women with medical cesarean indications

**Fig. 3:**
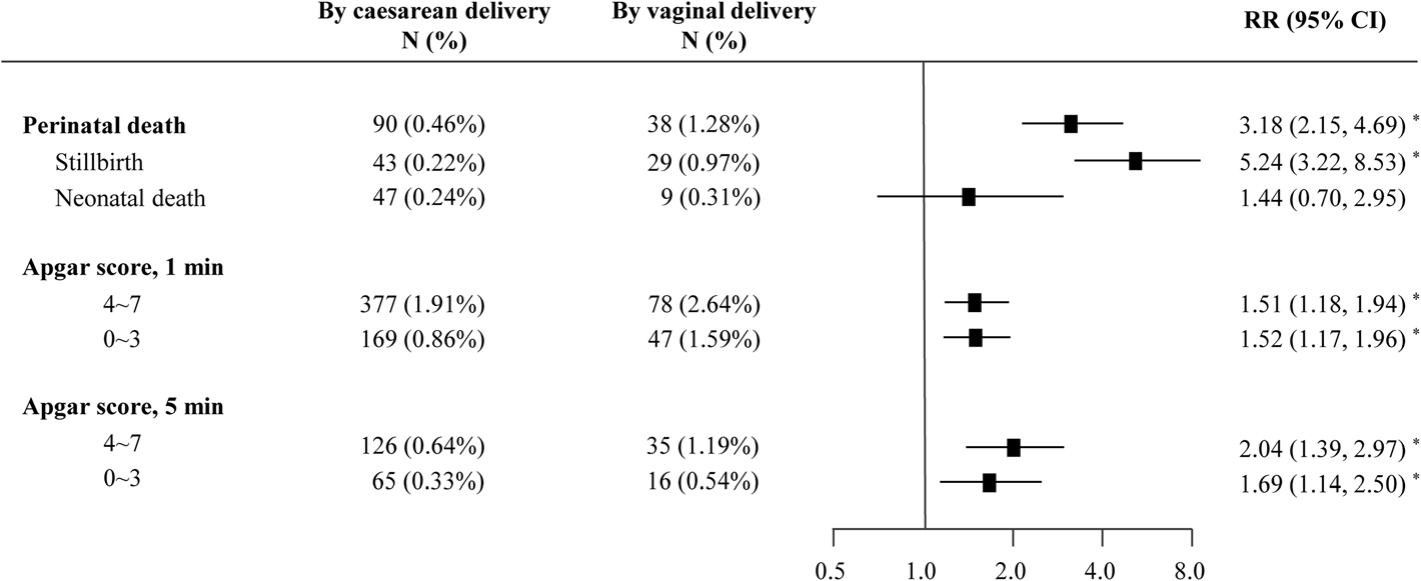
Incidence of adverse pregnancy outcomes of different delivery modes among mothers with CD indications

Considering yet no signs of improvement on the absolute percentage (13.10%) of vaginal delivery among women with medical cesarean indications from 2012 to 2019, it is critical to evaluate whether unrecommended delivery mode (vaginal delivery) raise the risks of adverse pregnancy outcomes. Log-binomial regression analysis showed that vaginal delivery was associated with a higher incidence rate of perinatal death (adjusted RR, 3.18; 95% CI, 2.15–4.69; *P* < 0.001) among women with medical indications. Being more specific, vaginal delivery increased the risk only for stillbirth, known as intrapartum fetal death (adjusted RR, 5.24; 95% CI, 3.22–8.53; *P* < 0.001), whereas the odds of neonatal death did not differ significantly between vaginal delivery and CD (adjusted RR, 1.44; 95% CI, 0.70–2.95). Additionally, vaginal delivery was significantly associated with lower Apgar score at 1 min after childbirth, when compared with CD (adjusted RR, 1.51, 95% CI, 1.18–1.94; adjusted RR, 1.52, 95% CI, 1.17–1.96, respectively). Similar association was observed for Apgar score at 5 min after childbirth (adjusted RR, 2.04, 95% CI, 1.39–2.97; adjusted RR, 1.69, 95% CI, 1.14–2.50, respectively) (Fig. 3). The above results revealed a higher risk of multiple adverse pregnancy outcomes among infants delivered vaginally by women who had medical indications, which suggested the medical necessity of CD for indicated women.

### Risks of adverse pregnancy outcomes elevated for deliveries with pregnancy complications and macrosomia who delivered by unrecommended delivery mode

Among the 14 medical cesarean indications, pregnancy complications occupied the highest proportion (*n* = 5530, 24.32%), followed by scarred uterus (*n* = 4719, 20.75%), complicated with tumor (*n* = 2403, 10.57%), placenta previa (*n* = 2252, 9.90%), abnormal fetal position (*n* = 1708, 7.51%), twin or multiple pregnancies (*n* = 1688, 7.42%), fetal macrosomia (*n* = 1305, 5.74%), and the other indications with a proportion < 5% (Additional file 3: Table S1). Besides, there were 1387 (6.10%) women with two or more CD indications. We then recorded 2980 (13.10%) cases of vaginal delivery in women with indications. Compared with other specific indications, women with fetal distress (3.76%), placental abruption (7.32%), pregnancy complications (34.70%), fetal macrosomia (10.84%), genital trace infects (0.74%), and combining with tumor (16.34%) had a higher proportion of vaginal delivery, the uncommented delivery mode (Additional file 3: Table S1).

Association analysis was further conducted to investigate whether vaginal delivery was associated with a higher risk of adverse pregnancy outcomes in each specific indication-subgroup with significant high proportion of uncommented delivery mode (Additional file 6: Figure S4). In particular, among women with pregnancy complications, the incidence rate of stillbirth in infants who delivered vaginally was 7.55 times higher than that of infants born by CD (adjusted RR, 7.55, 95% CI 2.59–21.98). In the fetal macrosomia subgroup, the risks of lower Apgar score at 1 min (adjusted RR, 8.99, 95% CI 1.74–46.55; adjusted RR, 6.81, 95% CI 1.34–34.50) were observed for women delivered vaginally as compared with those who had CD. The risk for perinatal death could not be estimated because there were no incidence cases for this outcome. For complicated with tumor subgroup, vaginal delivery was associated with a lower Apgar score at 5 min, when pregnant women were complicated with tumor. We observed null associations between delivery mode and adverse pregnancy outcomes in other indication-specific subgroups.

**Fig. 4:**
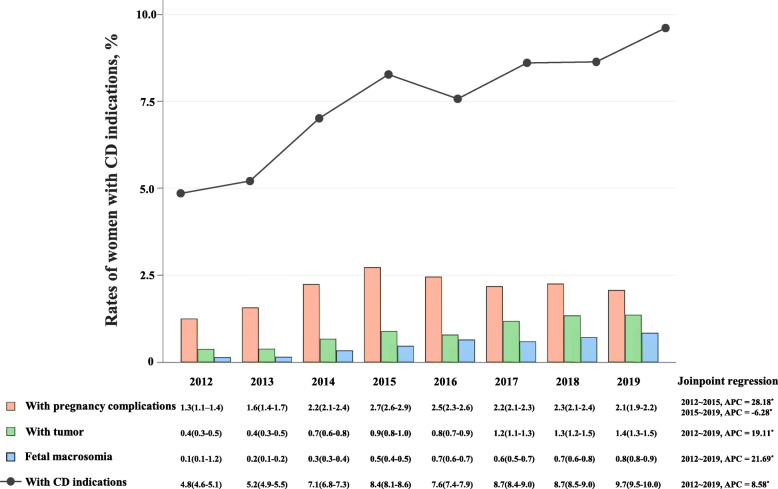
Secular trends in the incidence rate of each indication among the general population from 2012 to 2019, by year. Gray circles: annual incidence rates of the overall CD indications; orange blocks: annual incidence rates of women with pregnancy complications; green blocks: annual incidence rates of women with tumor; blue blocks: annual incidence rates of fetal macrosomia. The annual incidence rate and its 95% CI were listed below. Annual percentage change (APC) of the incidence rates of each indications estimated by joinpoint regression models was shown

In the former steps, we identified that women with pregnancy complications (includes tumor)/fetal macrosomia had a higher proportion of vaginal delivery, which delivery mode obviously increased the incidence risk of adverse pregnancy outcomes. Then, a connected-line plot by year was generated to visualize if any growth trend in the incidence rate of the above 3 indications. Figure 4 shows that the rate of women with pregnancy complications increased from 1.3% (1.1–1.4%) in 2012 to 2.1% (1.9–2.2%) in 2019. The peak was highest in 2015. Joinpoint analysis revealed two distinct segments: an initial period of growth (APC, 28.18 for 2012–2015; *P* < 0.001) and a plateau period (APC, − 6.28 for 2016–2019; *P* < 0.001). For the rate of women with tumor, a noteworthy increase of vaginal delivery rate was observed from 0.4% (0.3–0.5%) in 2012 to 1.4% (1.3–1.5%) in 2019 (APC, 19.11; *P* < 0.001). The rate of fetal macrosomia also increased considerably over time, from 0.1% (0.1–1.2%) in 2012 to 0.8% (0.8–0.9%) in 2019 (APC, 21.69; *P* < 0.001). To sum up, the overall rate of the 14 medical indications reached its peak in 2019 (9.7%), nearly 2 times higher than that in 2012, 4.8% (4.6–5.1%).

## Discussion

### Principal findings

The present study demonstrated the decreasing trend of overall CD rate over the period of 8 years in Jiangsu, China. When classified by with or without CD indications, we observed that the descend trend was largely driven by decline of the CD rate among women without indications, while the CD rate of women with indications has maintained approximate 87% steadily, with over 13% women choosing vaginal delivery each year. Association analysis among women with indications found that vaginal delivery raised the risks of adverse pregnancy outcomes. The effect was particularly prominent in deliveries with pregnancy complication (including tumor) and fetal macrosomia, of which incidence rates gradually increased during the study period.

In this data source with all of the deliveries from the 11 sampled hospitals in Jiangsu province, estimates of cesarean rates (50.93%) were substantially higher than the previous national census, which reported the national CD rate < 40% from 2008 to 2018 [10]. With this respect, our study stated Jiangsu as a province where CD is the predominant delivery mode in medical facilities, and the rate ranks by the front of the country [7, 16]. In our study, the significant decreases in cesarean rate are observed from 2012 to 2019 in this province with high-baseline rate in 2012. The decline in the cesarean rate was most pronounced in women without medical cesarean indications, with a reduction of 10% across the study period. Since the reported alarming CD rate in 2007–2008 [6], efforts has been made to cut down the rising tendency at the state level, such as the national Baby Friendly Hospital program from 2014 to 2016 [17] and the government initiatives on strictly controlling cesarean section rate and safeguarding maternal and child health since 2017 [18]. The implementation of the two-child policy also prompts the primiparous women to choose vaginal delivery given the potential adverse consequences of CD procedure in future pregnancies [11, 12, 19]. Other social factors also contribute to the increase in the vaginal delivery rate, for instance, social norms and hospital promotion of vaginal delivery, encouragement of the health care providers, vaginal birth after cesarean (VBAC) as a safe option for multiparas, the implementation of painless childbirth, and elimination of financial incentives. Altogether, reforms in family planning policy and powerful social efforts to promote vaginal birth have greatly changed the culture of delivery decision-making, leading to a drop of the overall CD rates.

In addition to the social consensus and policy initiatives, regional policy brings restraining effects to overall CD rates to different extent. Apparent decline in the overall CS rate was observed in Guangzhou, the largest city in southern China, after the implementation of a two-stage intervention package (October 2010–September 2014 and October 2014–December 2016). This intervention package was launched by the Health Commission of Guangzhou Municipality and included programs for population health education, skills training for healthcare professionals, equipment and technical support for local healthcare facilities, and capacity building for the maternal near-miss care system [8]. The overall CD rate in 2016 was 35.0%, with a reduction of 17% across the intervention stages. A similar CD rate (36.1%) was observed in 2014 after an intervention combined three antenatal care models was introduced in Shanghai, the largest city in eastern China [9]. Also, the CD rate in central and northwest China appeared a downward tendency in the recent years (more pronounced in nonessential cesarean deliveries) and was estimated as 38.9% in 2016 [11]. Obviously, the overall CD rate (49.76% in 2016–2019) in Jiangsu province was higher than that of the above areas. Strategies with rigorous design and formal evaluation are needed to further reduce the nonessential CD rate in this area.

Another unanticipated finding was the considerable percentage (13%) of absence of c-section among women with medical cesarean indications, along with no obvious improvement during the observation period. It revealed the insufficient cesarean service to women in need (with medical cesarean indications) in Jiangsu, China. In terms of CD rate, few studies have estimated it by specific medical indication, but instead using Robson classification system to divide women having CD according to their obstetric characteristics [20] (we also analyzed the CD rates according to the Robson 10 group criteria, shown in Additional file 7: Table S3). Some studies focused on identifying the proportion of cesarean sections for women in need in resource-poor settings [21, 22], where more than half of the CD procedures were not done for life-saving conditions. In the current study, we intended to identify the proportion of CD for women in need in Jiangsu, to explore the present existing obstetric problem in the province with a high CD rate.

The decision of performing CD primarily lies on what is the best mode for saving lives of mother and child. Classification system based on medical CD indications answers why the c-section being performed. However, classification system with unclear definitions for medical indications often results in questionable inter-rater reproducibility [23]. In many classification systems [24, 25], the main indications for CD are nonreassuring fetal status, previous cesarean, malpresentation, cephalopelvic disproportion, and nonprogress of labor. Other discussion of cesarean section revolves mainly around the division of the medical indications into absolute and relative indications [11, 26, 27]. In this study, each woman was classified into one absolute indication-specific group (woman with > 1 indications was grouped into each indication and therefore appear more than once in the study) to avoid collinearity and further to accurately estimate the necessity and validity of the medical indications. In addition to the mentioned obstetric indicators, severe pregnancy complications were brought into the indication system in our study, which were not definitely annotated in the previous guidelines or criteria [11, 26, 27]. In accordance with the present results, previous studies have demonstrated that cesarean section was advocated for various pregnancy complications, such as congenital or acquired cardiac disease [28, 29], idiopathic thrombocytopenic purpura and obstetric cholestasis [30], and pre-eclampsia [31]. Also, some studies indicated that surgical management of uterine fibroids and cervical malignancy at cesarean section may be a safe option with careful case selection [32, 33]. In the meantime, the implementation of the two-child policy causes a noteworthy increase in absolute numbers of women with advanced maternal age, which leads to the rapid growth of the incidence of pregnancy complications. These conditions together call for an explicit indication list on the cesarean section consensus.

Studies have concluded that morbidity and mortality increased significantly in the fetal macrosomia weighing 4500 g or greater [34, 35]. CD is considered as a practicable solution for reducing the risk of neonatal morbidity associated with fetal macrosomia. The issue is that weighing the newborn after delivery is the only way to accurately diagnose macrosomia. The prenatal diagnostic methods (i.e., assessment of maternal risk factors, clinical examination and ultrasonographic measurement) remain imprecise. The inaccurate prediction of macrosomia predisposes women to the improper delivery mode independent of actual birth weight [36, 37]. Although the diagnosis of fetal macrosomia is imprecise, prophylactic CD may be considered for suspected fetal macrosomia.

### Strengths and limitations

The most important strength of this study was the use of high-quality province-wide surveillance data to hierarchically assess the 8-year trends in cesarean rates according to having medical indications or not. During the observation period (after 2016), we observed an accumulation of women with medical indications, while the CD rates among these indicated women in need remained unimproved. Some major limitations of our study should be considered; most of all pertains to the representativeness of the selected hospitals. Nevertheless, the selected surveillance sites were randomly sampled within strata to ensure proportional representation of urban and rural populations across the province (eastern, central, northern, and western). However, considering hospitals’ medical quality, quality control ability, manpower, materials, and capital, only large hospitals with more than 1000 deliveries per year in each surveillance site were selected. Due to the medical quality disparity between large hospitals and primary hospital, this would be a limitation of the study’s generalizability to the rest of Jiangsu province.

## Conclusions

In conclusion, despite a statistically significant decrease in the CD rate being observed, the overall rate remained high. For women who had medical indications of CD, the vaginal delivery rate was considerable and the trend remained stable across the study period. Vaginal delivery among women with medical indications of CD was significantly associated with increased adverse pregnancy outcomes including stillbirth. Our study may have the following implications for future maternal policy-making in Jiangsu or other similar areas with high CD rates: (1) the modest decrease encourages a lot more needs to be done to reduce the CD rate in China; (2) composite interventions should be considered to improve the indicated CD rate and reduce the nonessential CD rate, instead of targeting the overall CD rate alone; and (3) pregnant women with underlying diseases and suspected fetal macrosomia should be provided individualized counseling about the risks and benefits of each delivery mode.

## Supplementary Information

**Additional file 1: Figure S1.** Location of the selective eleven hospitals in Jiangsu Province.

**Additional file 2: Figure S2.** Flowchart for selection of the participants included in the current analysis.

**Additional file 3: Table S1.** The cesarean delivery rate and vaginal delivery rate for women with specific medical cesarean indication.

**Additional file 4: Table S2.** Maternal characteristics according to mothers with or without CD indications, which were classified was indicated group and non-indicated group.

**Additional file 5: Figure S3.** Secular trend of required CD rate, by hospital level during 2012 and 2019, by year. Delivering hospital was categorized as grade 3A, grade 3B and grade 2A according to the Measures for the Administration of the Hospital Grade released by the Health Ministry.

**Additional file 6: Figure S4.** Associations between delivery mode and adverse pregnancy outcomes for each specific medical cesarean indication.

**Additional file 7: Table S3.** Number of deliveries and CD rates according to the Robson 10 group criteria.

## Data Availability

Data of the present research is available from the corresponding author on reasonable request.

## Abbreviations

WHO: World Health Organization
CD: Cesarean delivery
APC: Annual percentage change
CDMR: Cesarean delivery on maternal request rate
NMNMSS: National Maternal Near Miss Surveillance System
CPD: Cephalopelvic disproportion
SGA: Small for gestational age
LGA: Large for gestational age
RRs: Adjusted risk ratios
CIs: Confidence intervals
VBAC: Vaginal birth after cesarean
