# Supplementary figures and images for: The rates and medical necessity of cesarean delivery in China, 2012–2019: an inspiration from Jiangsu
Source: BMC Med. 2021 Jan 25;19:14. doi: 10.1186/s12916-020-01890-6 (PMC7831243; doi:10.1186/s12916-020-01890-6)

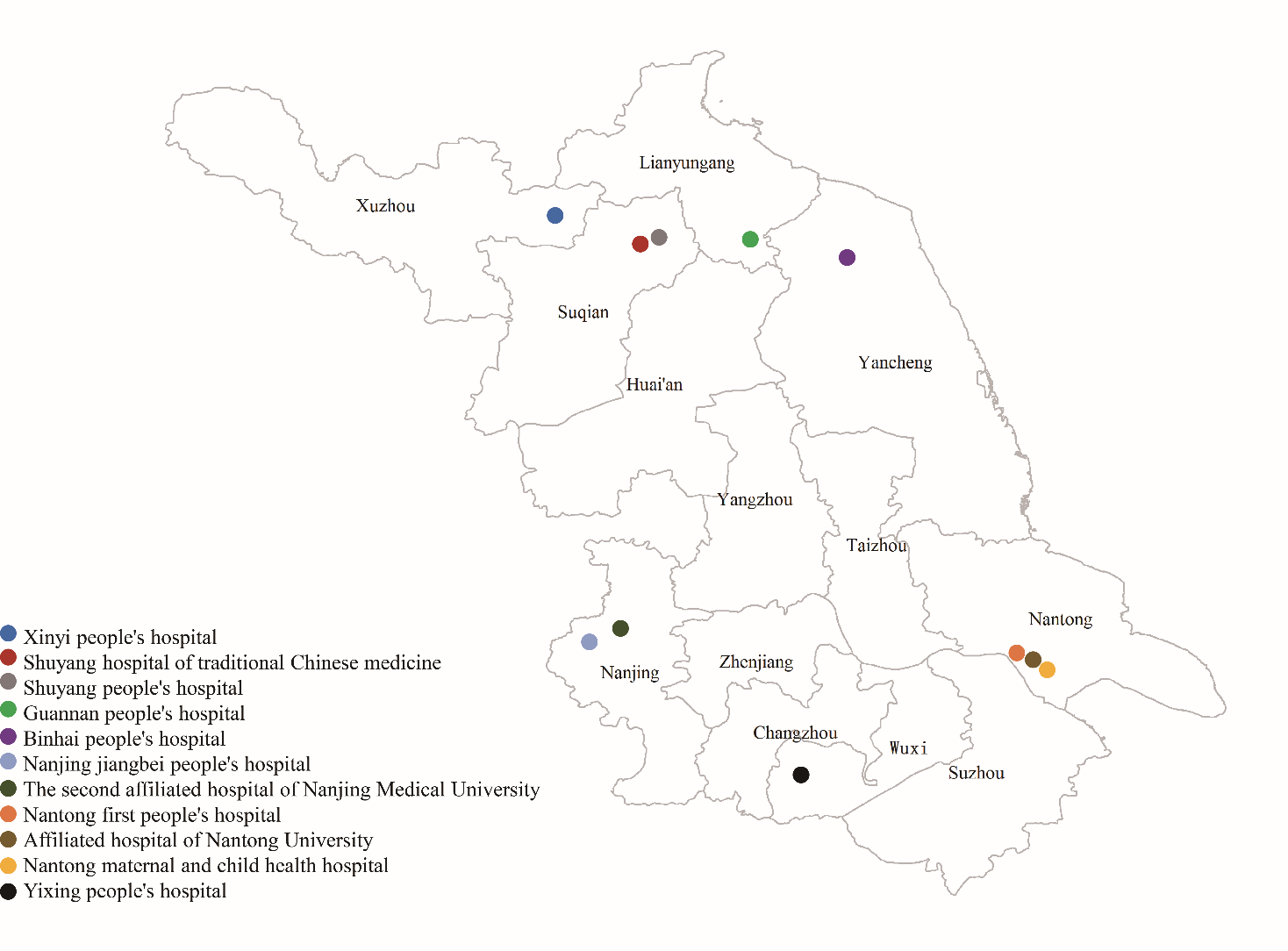

Supplement: Supplementary file 1 — Additional file 1: Figure S1. Location of the selective eleven hospitals in Jiangsu Province. [file 12916_2020_1890_MOESM1_ESM.tif]

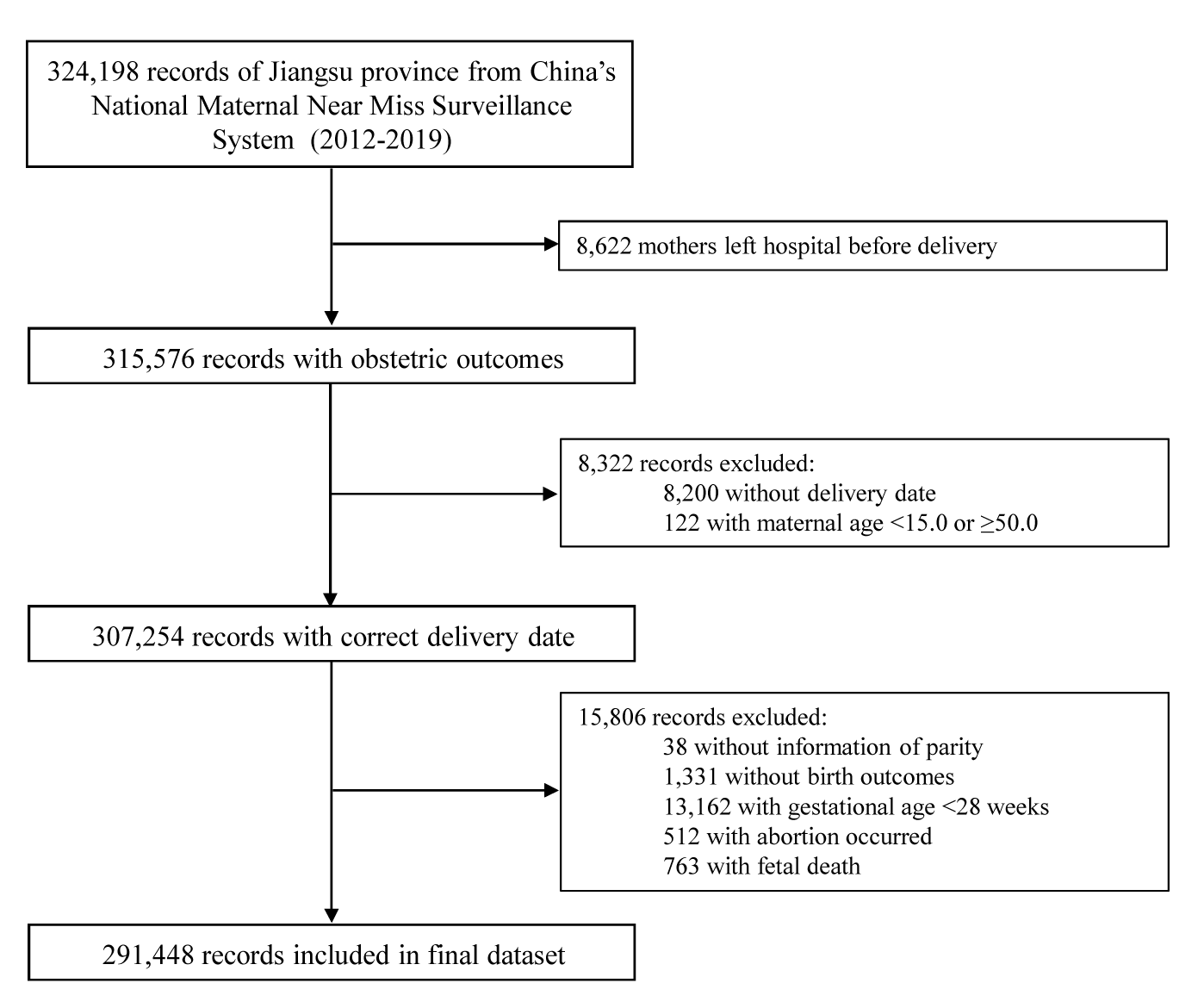

Supplement: Supplementary file 2 — Additional file 2: Figure S2. Flowchart for selection of the participants included in the current analysis. [file 12916_2020_1890_MOESM2_ESM.tif]

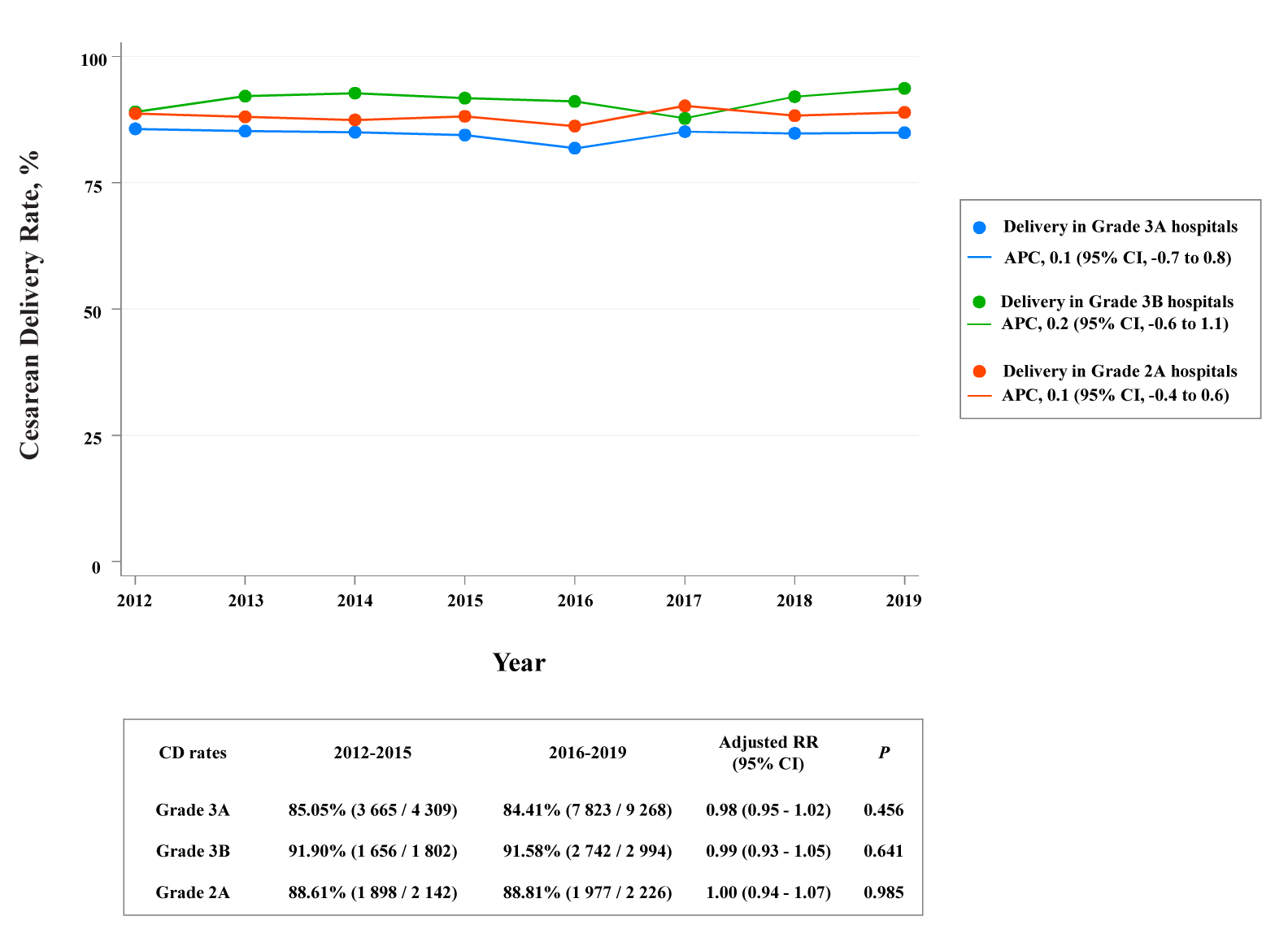

Supplement: Supplementary file 5 — Additional file 5: Figure S3. Secular trend of required CD rate, by hospital level during 2012 and 2019, by year. Delivering hospital was categorized as grade 3A, grade 3B and grade 2A according to the Measures for the Administration of the Hospital Grade released by the Health Ministry. [file 12916_2020_1890_MOESM5_ESM.tif]
