# Supplementary material for: The rates and medical necessity of cesarean delivery in China, 2012–2019: an inspiration from Jiangsu
Source: BMC Med. 2021 Jan 25;19:14. doi: 10.1186/s12916-020-01890-6 (PMC7831243; doi:10.1186/s12916-020-01890-6)
Supplement: Supplementary file 3 — Additional file 3: Table S1. The cesarean delivery rate and vaginal delivery rate for women with specific medical cesarean indication. [file 12916_2020_1890_MOESM3_ESM.docx]

| **Table S1. The cesarean delivery rate and vaginal delivery rate for women with specific medical cesarean indication.** | | | |  |
| --- | --- | --- | --- | --- |
| CD indications | Overall | By vaginal delivery | By caesarean delivery | *P* |
|  | N (%) | N (%) | N (%) |  |
|  | 22 741 (100.00) | 2 980 (13.10) | 19 761 (86.90) |  |
| Fetal distress | 478 (2.09) | 112 (3.76) | 366 (1.85) | <0.001 |
| Cephalopelvic disproportion | 33 (0.15) | 0 | 33 (0.17) | 0.026 |
| Scarred uterus | 4 719 (20.75) | 269 (9.03) | 4 450 (22.52) | <0.001 |
| Abnormal fetal position | 1 708 (7.51) | 102 (3.42) | 1 606 (8.13) | <0.001 |
| Placenta previa | 2 252 (9.90) | 118 (3.96) | 2 134 (10.80) | <0.001 |
| Twin or multiple pregnancies | 1 688 (7.42) | 186 (6.24) | 1 502 (7.60) | 0.008 |
| Omphaloproptosis | 24 (0.11) | 3 (0.10) | 21 (0.11) | 0.930 |
| Placental abruption | 897 (3.94) | 218 (7.32) | 679 (3.44) | <0.001 |
| Pregnancy complications | 5 530 (24.32) | 1 034 (34.70) | 4 496 (22.75) | <0.001 |
| Fetal macrosomia | 1 305 (5.74) | 323 (10.84) | 982 (4.97) | <0.001 |
| Birth canal malformation | 202 (0.89) | 33 (1.11) | 169 (0.86) | 0.354 |
| Varicose vulvar veins | 26 (0.11) | 5 (0.17) | 21 (0.11) | 0.171 |
| Genital tract infects | 89 (0.39) | 22 (0.74) | 67 (0.34) | 0.001 |
| Combined with tumor | 2 403 (10.57) | 487 (16.34) | 1 916 (9.70) | <0.001 |
| With two or more CD indications | 1 387 (6.10) | 68 (2.28) | 1 319 (6.67) | <0.001 |
