# Supplementary material for: The rates and medical necessity of cesarean delivery in China, 2012–2019: an inspiration from Jiangsu
Source: BMC Med. 2021 Jan 25;19:14. doi: 10.1186/s12916-020-01890-6 (PMC7831243; doi:10.1186/s12916-020-01890-6)
Supplement: Supplementary file 4 — Additional file 4: Table S2. Maternal characteristics according to mothers with or without CD indications, which were classified was indicated group and non-indicated group. [file 12916_2020_1890_MOESM4_ESM.docx]

| **Table S2**. Maternal characteristics according to mothers with or without CD indications, which were classified was indicated group and non-indicated group. | | | | |
| --- | --- | --- | --- | --- |
|  | All N (%) | Non-indicated group N (%) | Indicated group N (%) | *P* |
| Overall | 291 448 | 268 707 (92.20) | 22 741 (7.80) |  |
| Parity |  |  |  |  |
| Nullipara | 162 122 (56.66) | 154 092 (57.35) | 11 030 (48.50) | <0.001 |
| Multipara | 126 326 (43.34) | 114 615 (42.65) | **11 711 (51.50)** |  |
| Maternal age |  |  |  |  |
| 15~24 | 79 953 (27.43) | 75 987 (28.28) | 3 966 (17.44) | <0.001 |
| 25~29 | 133 494 (45.80) | 124 035 (46.16) | 9 459 (41.59) |  |
| 30~34 | 54 596 (18.73) | 48 663 (18.11) | **5 933 (26.09)** |  |
| ≥35 | 23 405 (8.03) | 20 022 (7.45) | **3 383 (14.88)** |  |
| Level of delivering hospital | | |  |  |
| Grade 3A | 144 056 (49.43) | 130 479 (48.56) | **13 577 (59.70)** | <0.001 |
| Grade 3B | 86 798 (29.78) | 82 002 (30.52) | 4 796 (21.09) |  |
| Grade 2A | 60 594 (20.79) | 56 226 (20.92) | 4 368 (19.21) |  |
| Maternal education |  |  |  |  |
| Graduate or above | 121 956 (41.92) | 111 099 (41.42) | **10 857 (47.82)** | <0.001 |
| High school | 116 966 (40.20) | 109 290 (40.75) | 7 676 (33.81) |  |
| Middle school | 48 528 (16.68) | 44 858 (16.72) | 3 670 (16.16) |  |
| Primary school or illiteracy | 3 477 (1.20) | 2 976 (1.11) | **501 (2.21)** |  |
| Mode of delivery |  |  |  |  |
| Vaginal delivery | 143 002 (49.07) | 140 022 (52.11) | 2 980 (13.10) | <0.001 |
| Caesarean delivery | 148 446 (50.93) | 128 685 (47.89) | **19 761 (86.90)** |  |
