# Supplementary material for: The rates and medical necessity of cesarean delivery in China, 2012–2019: an inspiration from Jiangsu
Source: BMC Med. 2021 Jan 25;19:14. doi: 10.1186/s12916-020-01890-6 (PMC7831243; doi:10.1186/s12916-020-01890-6)
Supplement: Supplementary file 7 — Additional file 7: Table S3. Number of deliveries and CD rates according to the Robson 10 group criteria. [file 12916_2020_1890_MOESM7_ESM.docx]

| **Table S3. Number of deliveries and CD rates according to the Robson 10 group criteria.** | | | | |
| --- | --- | --- | --- | --- |
| Group | Robson Classification | Overall  N (%) | By vaginal delivery N (%) | By caesarean delivery N (%) |
|  | Total | 291,448 (100) | 143,002 (49.07) | 148,446 (50.93) |
| Group 1 | Nulliparous women with single cephalic pregnancy, ≥37 weeks gestation in spontaneous labour | 84,297 (28.92) | 84,297 (58.95) | 0 (0.00) |
| Group 2 | Nulliparous women with single cephalic pregnancy, ≥37 weeks gestation who either had labour induced or were delivered by caesarean section before labour | 65,133 (22.35) | 0 (0.00) | 65,133 (43.88) |
| Group 3 | Multiparous women without a previous uterine scar, with single cephalic pregnancy, ≥37 weeks gestation in spontaneous labour | 50,024 (17.16) | 50,024 (34.98) | 0 (0.00) |
| Group 4 | Multiparous women without a previous uterine scar, with single cephalic pregnancy, ≥37 weeks gestation who either had labour induced or were delivered by caesarean section before labour | 59,887 (20.55) | 0 (0.00) | 59,887 (40.34) |
| Group 5 | All multiparous women with at least one previous uterine scar, with single cephalic pregnancy, ≥37 weeks gestation | 4,440 (1.52) | 234 (0.16) | 4,206 (2.83) |
| Group 6 | All nulliparous women with a single breech pregnancy | 5,253 (1.80) | 327 (0.23) | 4,926 (3.32) |
| Group 7 | All multiparous women with a single breech pregnancy, including women with previous uterine scars | 3,101 (1.06) | 316 (0.22) | 2,785 (1.88) |
| Group 8 | All women with multiple pregnancies, including women with previous uterine scars | 4,962 (1.70) | 672 (0.47) | 4,290 (2.89) |
| Group 9 | All women with a single pregnancy with a transverse or oblique lie, including women with previous uterine scars | 343 (0.12) | 38 (0.03) | 305 (0.21) |
| Group 10 | All women with a single cephalic pregnancy <37 weeks gestation, including women with previous scars | 14,008 (4.81) | 7,094 (4.96) | 6,914 (4.66) |
